# Development of dim-light vision in the nocturnal reef fish family Holocentridae. II: Retinal morphology

**DOI:** 10.1242/jeb.244740

**Published:** 2022-09-08

**Authors:** Lily G. Fogg, Fabio Cortesi, David Lecchini, Camille Gache, N. Justin Marshall, Fanny de Busserolles

**Affiliations:** 1 Queensland Brain Institute, The University of Queensland, Brisbane, Queensland 4072, Australia; 2 PSL Research University, EPHE-UPVD-CNRS, UAR3278 CRIOBE, 98729 Papetoai, Moorea, French Polynesia; 3 Laboratoire d'Excellence “CORAIL”, Paris 75006, France

**Keywords:** Ontogeny, Multibank retina, Teleost fish, Retinal structure, Histology

## Abstract

Ontogenetic changes in the habitats and lifestyles of animals are often reflected in their visual systems. Coral reef fishes start life in the shallow open ocean but inhabit the reef as juveniles and adults. Alongside this change in habitat, some species also change lifestyles and become nocturnal. However, it is not fully understood how the visual systems of nocturnal reef fishes develop and adapt to these significant ecological shifts over their lives. Therefore, we used a histological approach to examine visual development in the nocturnal coral reef fish family, Holocentridae. We examined 7 representative species spanning both subfamilies, Holocentrinae (squirrelfishes) and Myripristinae (soldierfishes). Pre-settlement larvae showed strong adaptation for photopic vision with high cone densities and had also started to develop a multibank retina (i.e. multiple rod layers), with up to two rod banks present. At reef settlement, holocentrids showed greater adaptation for scotopic vision, with higher rod densities and higher summation of rods onto the ganglion cell layer. By adulthood, they had well-developed scotopic vision with a highly rod-dominated multibank retina comprising 5–17 rod banks and enhanced summation of rods onto the ganglion cell layer. Although the ecological demands of the two subfamilies were similar throughout their lives, their visual systems differed after settlement, with Myripristinae showing more pronounced adaptation for scotopic vision than Holocentrinae. Thus, it is likely that both ecology and phylogeny contribute to the development of the holocentrid visual system.

## Abstract (precis)

**Summary:** Coral reef fishes in the family Holocentridae remodel their retina at the cellular level to adapt to a nocturnal lifestyle during development.

## INTRODUCTION

Vision is important to the behaviour and survival of most vertebrates (Cronin et al., 2014). Owing to the broad range of habitats and light environments that they experience, marine fishes show great diversity in their visual adaptations. These adaptations are reflected at the cellular level in the structure and organisation of their eye and retina (Walls, 1942; de Busserolles et al., 2020; Cortesi et al., 2020). The retina has 4 key cellular strata (in order of neural processing): the photoreceptor layer (PRL), outer nuclear layer (ONL), inner nuclear layer (INL) and ganglion cell layer (GCL). The PRL and ONL house the outer segments (OS) and nuclei of photoreceptors, respectively, of which there are two main types: rods and cones (Lamb, 2013). Rods usually mediate scotopic (dim light) vision, while cones mediate photopic (bright light) and colour vision, and are divided into single and double cones (i.e. two fused single cones). The synaptic terminals of rods and cones are contained within the outer plexiform layer (OPL) where they communicate with the next cellular layer, the INL.

The INL contains the nuclei of interneurons, such as bipolar, horizontal and amacrine cells, and their synapses are located within the inner plexiform layer (IPL), which represents the primary stage of opponent processing for colour vision (Baden and Osorio, 2019). Finally, visual signals are summated in the GCL, which sets the limits of the luminous sensitivity of the eye (i.e. more rods summating onto a single GC increases sensitivity) and spatial resolving power (i.e. acuity) (Warrant, 2004). Importantly, the density and distribution of the different neural cells are usually heterogenous across the retina. Regions of the retina with high densities of a particular cell type, i.e. retinal ‘specialisations’, provide higher acuity and/or sensitivity in a specific part of an animal's visual field (Collin and Pettigrew, 1989). Retinal specialisations often facilitate specific behavioural tasks, such as feeding or predator avoidance (Collin and Pettigrew, 1988a; Luehrmann et al., 2020; de Busserolles et al., 2021).

In general, differences in the organisation and densities of the retinal cell types correlate well with ecological demands (Shand, 1997; Stieb et al., 2016; Luehrmann et al., 2020). For instance, fishes that are predominantly active in dim light (e.g. those with a deep-sea habitat or nocturnal lifestyle) have evolved a shared array of cellular adaptations to enhance the sensitivity of their eyes, including high rod densities and low cone densities (Pankhurst, 1989; Shand, 1994b), high summation of rods onto GC (Shand, 1994b; de Busserolles et al., 2020, 2021) and thick PRL (with longer rods) (Wagner et al., 1998). Several species have pushed scotopic adaptations to an extreme level by evolving a pure rod retina (Munk, 1966) or a retina with multiple layers of rods (known as a multibank retina) (McFarland, 1991; de Busserolles et al., 2021), or by combining the characteristics of both rods and cones into a single photoreceptor cell (known as transmutation) (de Busserolles et al., 2017). Although some of these adaptations are relatively common, little is known about their development.

Over ontogeny, many marine fishes experience significant ecological shifts. As larvae, most marine fishes inhabit a bright and broad-spectrum light environment (Boehlert, 1996) in the upper epipelagic ocean where they consume (zoo)plankton (Job and Bellwood, 2000; Helfman et al., 2009). As they grow older and become juveniles and adults, they may switch to a very different habitat (pelagic, estuarine, reef, deep-sea), diet (planktivory, carnivory, herbivory, corallivory) and/or diel activity pattern (diurnal, nocturnal, crepuscular) (King and McFarlane, 2003; Helfman et al., 2009). These ecological shifts and consequent changes to the light environment are thought to be the main drivers of visual development in marine fishes (Carleton et al., 2020; Musilova et al., 2021). As such, ontogenetic variation in the organisation and/or structure of their visual systems have previously been correlated with changes in diet (surgeonfishes: Tettamanti et al., 2019), diel activity patterns (several reef fish families: Shand, 1997), depth (lanternfishes, hoki, hake, roughy, oreodories: Pankhurst, 1987; Mas-Riera, 1991), habitat (goatfishes: Shand, 1994a; mackerel icefish: Miyazaki et al., 2011) and body morphology (winter flounder: Evans and Fernald, 1993).

For species that adopt bright environments, visual development is characterised by typical changes in the cellular architecture of the retina. Specifically, the retina is initially cone-dominated, and the densities of cones, INL cells and GC increase early in development and then decrease slightly (as retinal area expands), while rod densities undergo a minor increase (Fernald, 1990; Shand, 1997). Contrastingly, in fishes which adopt dim environments, visual development seems to be characterised by a more rapid and extreme version of these changes. For example, some deep-sea fishes seem to possess cones as larvae but progress to having only rods in adulthood (Bozzano et al., 2007; de Busserolles et al., 2014a; Lupše et al., 2021). However, most of the previous studies on visual development in fishes with dim habitats or lifestyles focused on deep-sea fishes. In contrast, how the visual system develops in nocturnal reef fishes is poorly understood [but see (Shand, 1997)].

Here, we investigated visual development at the cellular level in the nocturnal reef fish family, Holocentridae. Holocentridae comprises two subfamilies: Holocentrinae (squirrelfishes) and Myripristinae (soldierfishes). As larvae, both subfamilies inhabit the upper pelagic ocean and feed on zooplankton (Tyler et al., 1993; Sampey et al., 2007). During the transition to juvenile life, most holocentrids migrate to a shallow tropical coral reef habitat (Nelson, 1994) and adopt a nocturnal lifestyle feeding on benthic crustaceans (Holocentrinae) or zooplankton in the water column (Myripristinae) (Gladfelter and Johnson, 1983; Greenfield, 2002; Greenfield et al., 2017). Recently, we examined the visual systems of adult holocentrids (de Busserolles et al., 2021). We found that they possess well-developed scotopic vision with a rod-dominated retina arranged into multiple banks. The complexity of their multibank retina resembles that of some deep-sea fishes, with up to 7 and 17 banks in Holocentrinae and Myripristinae, respectively (de Busserolles et al., 2021). Adults also have some level of photopic vision which is more pronounced in Holocentrinae than Myripristinae, with the presence of both single cones and double cones, all well organised into retinal specialisations (de Busserolles et al., 2021).

While the visual systems of adult holocentrids have been described in detail, their development is poorly understood. Hence, we used a histological approach to examine anatomical structure and cell densities in the retina at key ontogenetic stages (pre-settlement larvae, settlement larvae, settled juveniles and adults). We studied shallow reef-dwelling species from three genera (*Sargocentron*, *Neoniphon* and *Myripristis*) covering both subfamilies, as well as an adult for a deeper-dwelling species (*Ostichthys* sp.). We used this approach to address the following aims: (1) to assess how the holocentrid visual system changes as they shift from being predominantly active in bright light to dim light, and (2) to study the development of their deep-sea-like multibank retina.

## MATERIALS AND METHODS

### Animal collection and retinal tissue preservation

Details of all animals used in this study are given in Table S1. Animal collection and developmental staging followed methods outlined in greater detail in Fogg et al. (2022). Briefly, pre-settlement larvae were collected using light traps and settlement larvae were collected using a crest net (Lecchini et al., 2004; Besson et al., 2017). Settled juveniles were larvae caught in light traps which were allowed to metamorphose and further develop for 2 weeks in outdoor aquaria exposed to natural light. Adults were collected with either spearguns, pole and lines or clove oil and hand nets, or were sourced from a supplier, Cairns Marine (Cairns Marine Pty Ltd, Cairns, Australia; https://www.cairnsmarine.com/).

Fish collection and euthanasia followed procedures approved by the University of Queensland Animal Ethics Committee (QBI 304/16). Briefly, fish were first anesthetised by immersion in a solution of 0.2 ml clove oil per litre of seawater until respiration and all response to light and touch had ceased and were then euthanised by swift decapitation. All collections within Australia were conducted under a Great Barrier Reef Marine Park Permit (G17/38160.1) and Queensland General Fisheries Permit (180731) and all collections in French Polynesia were conducted in accordance with French regulations. Following euthanasia, all individuals were photographed adjacent to a ruler and their body size (total length and standard length) and eye diameter were subsequently measured from photographs using Fiji v.1.53c (National Institutes of Health, USA; Schindelin et al., 2012). Eyes were immediately enucleated, the cornea and lens removed, and the eye cup preserved in 4% paraformaldehyde [PFA; 4% (w/v) PFA in 0.01 mol l^−1^ phosphate-buffered saline (PBS), pH 7.4] depending on the analysis (see below for details).

### Histology

**Fig. 1.**
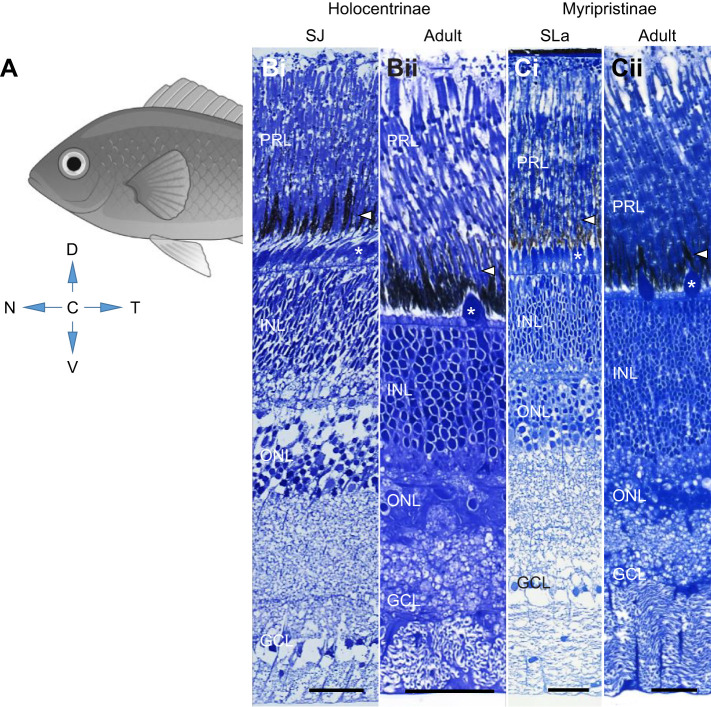
**Histological sampling of the retina.** (A) Schematic illustrating the locations of the dorsal (D), ventral (V), central (C), nasal (N) and temporal (T) regions of the retina in an intact fish. (B–C) Representative radial sections of the entire retina from different life stages in Holocentrinae (B) and Myripristinae (C), illustrating the different retinal layers for which cell densities were estimated. A representative rod and cone photoreceptor is indicated in each section by an arrow and an asterisk, respectively. PRL, photoreceptor layer; INL, inner nuclear layer; ONL, outer nuclear layer; GCL, ganglion cell layer; SLa, settlement larva; SJ, settled juvenile. Scale bars: 30 µm.

Retinal histology was conducted on PFA-fixed eyes from the following individuals: three pre-settlement larvae (*Sargocentron rubrum*, *n*=3), five settlement larvae (*Sargocentron microstoma*, *n*=1; *Myripristis berndti*, *n*=1; *Myripristis kuntee*, *n*=3), two settled juveniles (*Sargocentron rubrum*, *n*=2) and ten adults (*S. rubrum*, *n*=3; *S. microstoma*, *n*=1; *Sargocentron diadema*, *n*=1; *M. berndti*, *n*=2; *M. kuntee*, *n*=1; *Myripristis violacea*, *n*=1; *Ostichthys* sp., *n*=1). All animals were sampled in the light-adapted state except for the pre-settlement larvae and the adult *Ostichthys* specimen, which were dark adapted. To account for intraretinal variability (de Busserolles et al., 2021), 2 (dorsal and ventral) or 5 (dorsal, ventral, central, nasal and temporal) retinal regions were sampled for pre-settlement larvae and later stages, respectively (Fig. 1). Notably, for the *Ostichthys* sp., tissue quality was only sufficient to examine one region (ventral). Briefly, a small square of retina was dissected from each region, post-fixed in 2.5% glutaraldehyde and 2% osmium tetroxide, dehydrated in ethanol and/or acetone, and embedded in EPON resin (ProSciTech). All tissue processing was done in a BioWave Pro tissue processor (PELCO).

Radial 1-μm-thick retinal sections were cut with a glass knife on a Leica ultramicrotome (Ultracut UC6) and stained with a solution of 0.5% Toluidine Blue and 0.5% borax. Retinal sections were viewed with a 63× objective (oil, 1.4 numerical aperture, 0.19 mm working distance, 0.102 μm pixel^−1^) on a Zeiss Axio upright microscope (Imager Z1) and brightfield images acquired with Zeiss Axiocam 506 mono and 512 colour microscope cameras. Rod outer segment (ROS) length, PRL thickness, and whole retinal thickness were then measured from micrographs using Fiji. A body size range at which the full complement of banks (i.e. the maximum number of rod banks detected across all individuals/stages for each species) was reached was determined by comparing total length in individuals with the full complement to the maximal total length published in FishBase (Froese and Pauly, 2019, see: https://www.fishbase.se).

### Cell density estimations

Retinal cell densities were estimated from transverse retinal sections, a method widely employed for marine teleosts for over 50 years (Munk, 1965; Locket, 1980; Shand, 1997; Taylor et al., 2015). Cell densities were compared at different stages in the same species for Holocentrinae (*S. rubrum*), and in two species in the same genus for Myripristinae because of a limitation in the number of specimens at specific stages (settlement: *M. kuntee*, adult: *M. berndti*). However, to make sure that the data were comparable between the different species from Myripristinae, the densities in settlement *M. kuntee* were also compared to one settlement *M. berndti* and the densities in adult *M. berndti* were compared to one adult *M. kuntee* (Fig. S1). Briefly, using Fiji, images were cropped to obtain retinal strips of 250 μm (horizontal length) for lower-density cell types (i.e. cones and GCL cells) in adults, 100 μm for lower-density cell types in larvae, and 40 μm for higher-density cell types (i.e. ONL and INL cells) for all life stages. These counting frames were optimised by conducting trials with several frame sizes and taking the minimum frame size that produced counts ≥95% similar to those attained with the largest frame (assumed to be the most accurate). The number of cone OS, ONL nuclei, INL nuclei and GCL nuclei were counted for three sections per sample using the cell counter plugin in Fiji. Subsequently, counts were corrected for section thickness using Abercrombie's correction (Abercrombie, 1946) and the density of each retinal cell type per 0.01 mm^2^ of retina was calculated. Rod densities were calculated as the difference between ONL nuclei and cone OS densities, while rod:GCL summation was calculated by dividing the densities of rods by the densities of cells in the GCL (Shand, 1994a). Graphs throughout the manuscript were generated using GraphPad Prism software v.8.3.1 (www.graphpad.com).

## RESULTS

### Multibank retina structure

**Fig. 2.**
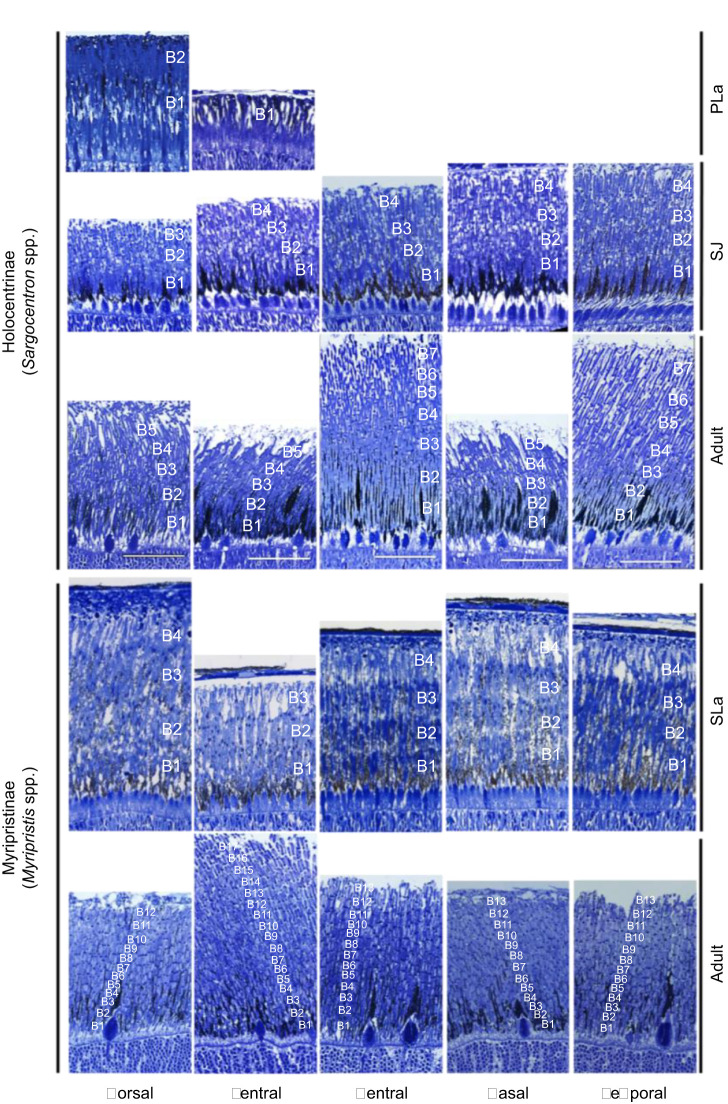
**Development of the multibank retina in different retinal regions in Holocentrinae and Myripristinae.** Representative radial sections of the retina in key retinal regions showing photoreceptor layer with multiple banks of rods at different ontogenetic stages in Holocentrinae (pre-settlement *Sargocentron rubrum*, settled juvenile *S. rubrum* and adult *S. diadema*) and Myripristinae (settlement larval *Myripristis kuntee* and adult *M. berndti*). Rod banks are numbered as B1−B*n*. Scale bars in central row of images are accurate for all images. PLa, pre-settlement larva; SJ, settled juvenile; SLa, settlement larva. Scale bars: 50 µm.

Retinal sections were taken at different life stages from species in each subfamily in Holocentridae to assess the structure of their multibank retina. In all species and stages, rods were arranged in banks in at least part of the retina. Moreover, rod banking increased with size/age. Pre-settlement larvae of *S. rubrum* (the only species obtained at this stage) had two rod banks in the dorsal retina but only one bank in the ventral retina (Fig. 2). In settled juveniles from Holocentrinae, this increased to 3 or 4 rod banks depending on the region, and in adults, increased to 5 banks in the dorsal, nasal and ventral regions and 7 banks in the temporal and central retina (Fig. 2). Settlement larvae of *M. kuntee* and *M. berndti* had 3–4 rod banks in all regions, while adults possessed 12–13 banks in all regions except the ventral retina, which had 17 banks (Fig. 2). Finally, the adult specimen of the deeper dwelling soldierfish, *Ostichthys* sp., had approximately 10 rod banks in the ventral retina (Fig. S2). Across the family, the full complement of banks was attained by the time fish reached 40–60% of maximal size.

In species from both subfamilies (Holocentrinae: *S. microstoma*; Myripristinae: *M. berndti*), the addition of rod banks between settlement larvae/settled juveniles and adults resulted in an increase in the PRL thickness (Table 1). The regions with the greatest increase in rod banks over ontogeny showed the greatest increase in PRL thickness and maximal PRL thickness matched maximal rod banking in adults. However, the ontogenetic increase in rod banking did not result in a linear increase in PRL thickness because of concurrent shortening of the ROS with age (Table 1).

**Table 1.**
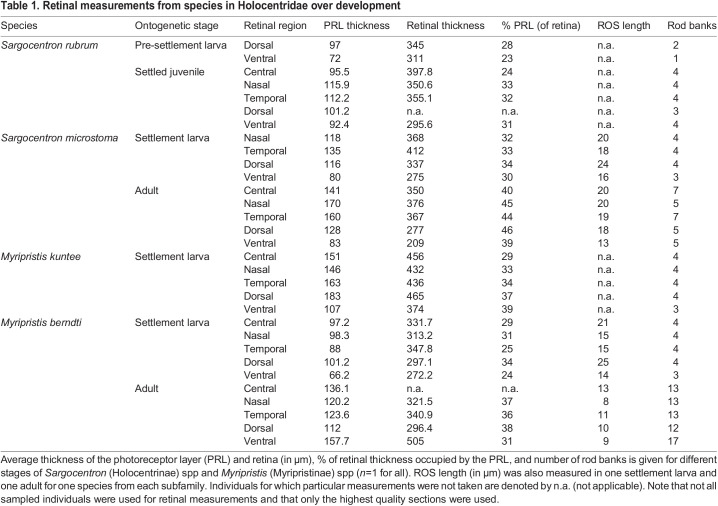
Retinal measurements from species in Holocentridae over development

### Retinal cell densities

**Fig. 3.**
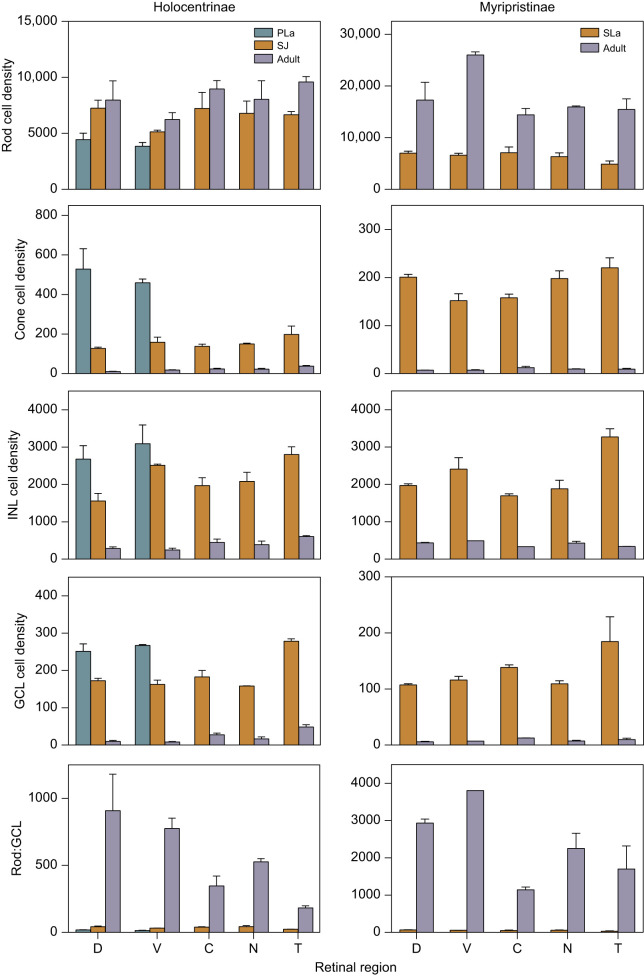
**Retinal cell densities in holocentrids over ontogeny.** Abercrombie-corrected densities of rods, cones, inner nuclear layer (INL) cells and ganglion cell layer (GCL) cells, and rod:GCL summation in the dorsal (D), ventral (V), central (C), nasal (N) and temporal (T) retina in Holocentrinae [*Sargocentron rubrum* pre-settlement larvae (*n*=3), settled juveniles (*n*=2) and adults (*n*=3)] and Myripristinae [*Myripristis kuntee* settlement larvae (*n*=3) and *M. berndti* adults (*n*=2)]. Cell densities are cells per 0.01 mm^2^ of retina presented as means±s.e.m. Green, pre-settlement larvae (PLa); orange, settlement larvae (SLa; Myripristinae) or settled juveniles (SJ; Holocentrinae); purple, adults. Cell measurements used for Abercrombie's correction are given in Table 2.

The densities of different retinal cell types (rods, cones, INL cells and GCL cells) were estimated in different regions of the retina for species in Holocentrinae (*S. rubrum*) and Myripristinae (*M. kuntee*, *M. berndti*) (Fig. 3). In *S. rubrum*, between pre-settlement larval and settled juvenile stages, mean cone, INL and GCL densities decreased across the retina, by 65–75%, 19–42% and 31–39%, respectively (% range for the different regions) (Fig. 3, Table 3). Concurrently, rod densities and rod:GCL summation increased in all regions, by 34–63% and 120–136%, respectively. Between settled juvenile and adult stages, cone, INL and GCL cell densities continued to decrease across the retina, by 81–92%, 77–90% and 83–95%, respectively. Concurrently, rod densities and rod:GCL summation further increased by 10–44% and 663–2073%, respectively.

**Table 2.**
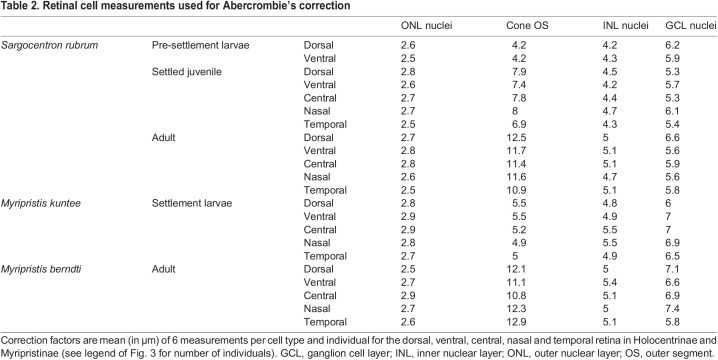
Retinal cell measurements used for Abercrombie's correction

**Table 3.**
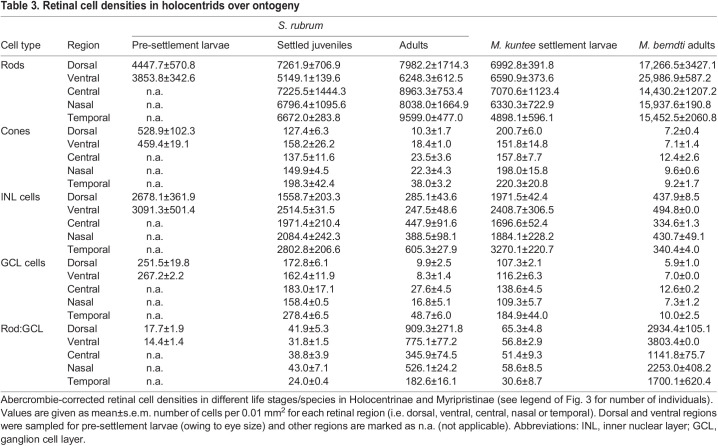
Retinal cell densities in holocentrids over ontogeny

A similar developmental pattern was observed in *Myripristis* spp. (Fig. 3, Table 3). Since cell densities were found to be similar between *M. kuntee* and *M. berndti* (Fig. S1), a comparison between stages was done using the two species to increase sample size. Between settlement and adulthood, cone, INL cell and GCL cell densities decreased across the retina by 92–96%, 77–90% and 90–95%, respectively. Concurrently, rod densities and rod:GCL summation increased by 104–294% and 2123–6592%, respectively.

Intraretinal shifts in peak cell densities were also found in all holocentrids examined (Fig. 3, Table 3). Around settlement, all species had higher cone, INL cell and GCL cell densities in the temporal retina. At adulthood, *S. rubrum* retained these peak densities in the temporal retina, while *Myripristis* spp. shifted its peak cone and GCL cell densities centrally, and its peak INL cell densities ventrally. Conversely, rod densities did not peak in the same regions for *S. rubrum* and *Myripristis* spp. at either stage (Fig. 3). Lastly, the highest densities for each cell type were similar around settlement, irrespective of subfamily, but by adulthood, *S. rubrum* had much lower peak rod densities and higher peak cone and GCL cell densities than *Myripristis* spp.

## DISCUSSION

### Development of the multibank retina

The multibank retina is one of the most common visual specialisations in deep-sea fishes, found in at least 38 families from across the teleost phylogeny (de Busserolles et al., 2020; Awaiwanont et al., 2001). Based on the few studies on multibank retina development, it appears that rod banks are added as fish grow (Locket, 1980; Pankhurst, 1987; Frohlich and Wagner, 1998; Wagner et al., 1998; Omura et al., 2003; Taylor et al., 2011, 2015), either continually (for mesopelagic fishes and one catadromous elopomorph species, *Anguilla japonica*), or until 20–47% of the maximal size is reached (for bathypelagic fishes) (Locket, 1980; Pankhurst, 1987; Frohlich and Wagner, 1998; Omura et al., 2003). Similarly to bathypelagic fishes, the present study showed that in holocentrids, banks were added as the fish grew (Fig. 2, Table S1), until they reached 40–60% of their maximal size. However, this may be found to be even earlier if more intermediate sizes were examined. Moreover, most banks were added after holocentrids settled on the reef and transitioned to a dimmer environment. Whether the addition of rod banks was driven by the exposure to dim light is still unknown. However, light environment has consistently been shown to be the dominant driver of visual adaptations in marine fishes (Shand et al., 2008; Cortesi et al., 2016; Luehrmann et al., 2018; Schweikert et al., 2018) and thus, represents a convincing possibility.

Further evidence for light environment as a driver of multibank retina development comes from an examination of intraretinal and interspecific variability in bank numbers. This variability has been reported in some adult deep-sea fishes (Locket, 1985; Denton and Locket, 1989) as well as some adult holocentrids (de Busserolles et al., 2021). Similarly, this study showed that the number of banks in adult holocentrids varied with both retinal region and species (Fig. 2). However, at earlier stages, rod banking did not vary greatly with either factor. Thus, the holocentrid multibank retina only became specialised later in life once they had adopted a nocturnal lifestyle. This implies that the multibank adaptation does not become fully active until maturity and/or under dim conditions.

Despite the prevalence of multibank retinas, their function remains a mystery. Two main non-mutually exclusive hypotheses have been proposed: (1) multibank retinas enhance luminous sensitivity (Frohlich and Wagner, 1998) and/or (2) they allow colour vision in dim light (Denton and Locket, 1989). Results from this study seem to support both ideas. Support for the sensitivity hypothesis comes from the co-localisation of peak rod:GCL convergence and peak rod banking in Myripristinae (Figs 2 and 3), suggesting that summation of visual signals is prioritised in their multibank retina. Conversely, support for the colour vision hypothesis comes from the co-localisation of peak INL cell densities with peak cone densities at settlement but peak rod densities in adulthood (Fig. 3). Given that the INL contains the nuclei of cells involved in the primary stages of opponent processing (Baden and Osorio, 2019), this potentially suggests a developmental switch in opponent processing of cone- to rod-derived signals. Although these are intriguing insights, future electrophysiological and behavioural studies are required to confirm the function of the multibank retina throughout ontogeny.

### Retinal cell densities over development

Most teleosts commence life with a pure cone retina, with rods added later (Evans and Fernald, 1990; Raymond et al., 1995). While most diurnal shallow-water fishes follow this developmental trajectory (Blaxter and Staines, 1970), it may be adjusted when they are faced with different ecological demands. For example, deep-sea or nocturnal fishes show more rapid and pronounced increases in rod densities and decreases in cone densities over development (Shand, 1997; Locket, 1980; Pankhurst, 1987; Bozzano et al., 2007). In line with their ecology, holocentrids followed a nocturnal pattern, rapidly decreasing cone densities and increasing rod densities (Fig. 3, Table 3). These retinal changes were particularly pronounced post-settlement, correlating with the timing at which holocentrids are thought to become nocturnal (Shand, 1994b). Moreover, the extent of developmental changes differed between the two subfamilies. At settlement, both subfamilies had similar visual systems. However, in adults, higher rod densities and lower cone densities were found in Myripristinae compared with Holocentrinae, similar to findings from retinal wholemounts (de Busserolles et al., 2021). Thus, Holocentrinae retained more of their photopic visual system, the reason for which requires further studies on their daytime activities. In summary, the holocentrid visual system is remodelled at the cellular level over development to suit their nocturnal lifestyle, while still maintaining some adaptation for daytime activity.

Shallow-water holocentrids are thought to have emerged from a deep-water existence and some of their extant relatives are still found at greater depths, down to 640 m (Yokoyama et al., 2008; Greenfield et al., 2017). Given this phylogenetic connection to the deep-sea, it is not surprising that some aspects of their visual development were comparable to deep-sea fishes while others more closely resembled shallow-water fishes. In terms of the cones, a steep decline in densities was evident during development (Fig. 3, Table 3) and the adult population was mainly composed of double cones, similar to the situation reported for some deep-sea fishes (Boehlert, 1979; Munk, 1990; de Busserolles et al., 2021). However, holocentrids retained cones in all retinal regions throughout life, whereas these are often lost at early developmental stages (Bozzano et al., 2007) or become restricted to certain retinal regions (Munk, 1990) in some deep-sea fishes. With respect to rods, adult holocentrids (particularly in Myripristinae) possessed peak densities that rival those of some deep-sea fishes (e.g. *M. berndti*: ∼2.6 million rods mm^−2^ vs. *Myctophum brachygnathum*: ∼2 million rods mm^−2^ and *Hoplostethus atlanticus*: ∼1.7 million rods mm^−2^) (Pankhurst, 1987; de Busserolles, 2013) and their maximal rod:GCL summation even exceeds that of many deep-sea species (e.g. *M. berndti*: 3800:1 vs. *Lampanyctodes* spp.: 2000:1 and *Chauliodus sloani*: 200:1) (Locket, 1980; Pankhurst, 1987). Finally, the developmental decrease in GCL cell densities in holocentrids is intermediate compared with the very steep decrease observed in deep-sea fishes (Locket, 1980; Pankhurst, 1987) and the more subtle change found in diurnal shallow-water species (Johns and Easter, 1975; Shand et al., 2000).

### Ontogenetic shifts in retinal specialisations

Retinal specialisations in teleosts usually reflect ecological demands (Collin and Pettigrew, 1988a,b; Luehrmann et al., 2020; de Busserolles et al., 2014b; Collin, 2008) and, accordingly, have been shown to shift during ontogeny (Shand et al., 2000; Tettamanti et al., 2019). This is also the case in the holocentrids. At settlement, all species had similar retinal specialisations (Fig. 3). The region with greater acuity (i.e. highest GCL cell densities) and better adaptation for bright light vision (i.e. highest cone densities) was found in the temporal retina. This area surveys the visual field immediately in front of the fish, which may help the larvae to see their small zooplankton prey in the brightly lit surface layers of the ocean (Kawamura et al., 1984; Shand et al., 2000). On the other hand, larval holocentrids showed the highest sensitivity (i.e. highest rod densities and rod:GCL ratio) in the dorsal retina, which surveys the visual field beneath the fish and may be used to spot predators coming from the dimmer waters below (Collin and Partridge, 1996).

After holocentrids have settled on the reef and adopted their nocturnal lifestyle, their retinal specialisations shift accordingly (Fig. 3). In adults, the regions with the highest acuity (i.e. highest GCL cell densities) were located temporally in Holocentrinae (i.e. looking forward) and ventro-temporally in Myripristinae (i.e. looking forward and upwards). These specialisations are likely to be linked to their nocturnal feeding ecologies. As benthic feeders, prey would be viewed in front of Holocentrinae when the mouth is angled towards the seafloor, while Myripristinae feed in the water column where food items usually occur in front of and above fishes [also see: de Busserolles et al. (2021)]. The regions which are best adapted for sensitivity (i.e. highest rod densities) overlapped with the regions of higher acuity in both subfamilies (Holocentrinae: central and temporal; Myripristinae: ventral) and so may also facilitate nocturnal feeding. Finally, the regions with the greatest adaptation for bright light vision (i.e. highest cone densities) were located temporally in Holocentrinae and centrally in Myripristinae, surveying the area in front of or lateral to the fish, respectively. Little is known about the daytime activities of holocentrids; however, these areas may be linked to social interactions and identification of safe havens for refuge during the day (Winn et al., 1964; Carlson and Bass, 2000).

### Conclusion

The holocentrid visual system adapted to life in dim light over ontogeny. At the morphological level, they increased rod banks from 1-2 to 5-17, adopted a rod-dominated retina and increased visual summation. Despite the early emergence of the multibank retina, substantial topographic specialisations in bank number were only evident after the transition to a dimmer environment. Together, this suggests that ecology drives visual development in Holocentridae. However, subfamily-specific differences in the degree of scotopic specialisation emerged over development (i.e. more rod banks, higher rod densities and greater summation in Myripristinae) and these were correlated with phylogenetic relatedness to deep-water representatives rather than ecology. This suggests that the development of the holocentrid retina may also be somewhat driven by phylogeny. Future studies on visual development in other nocturnal reef fishes, as well as other marine fish families with both shallow- and deep-water forms, such as Anomalopidae (flashlight fishes) and Engraulidae (anchovies), may provide further insights into the ecological and phylogenetic drivers of the development of dim-light vision.

## Supplementary Material

Supplementary information
