# Supplementary material for: Development of dim-light vision in the nocturnal reef fish family Holocentridae. II: Retinal morphology
Source: J Exp Biol. 2022 Sep 8;225(17):jeb244740. doi: 10.1242/jeb.244740 (PMC9482369; doi:10.1242/jeb.244740)
Supplement: Supplementary information [file jexbio-225-244740-s1.pdf]

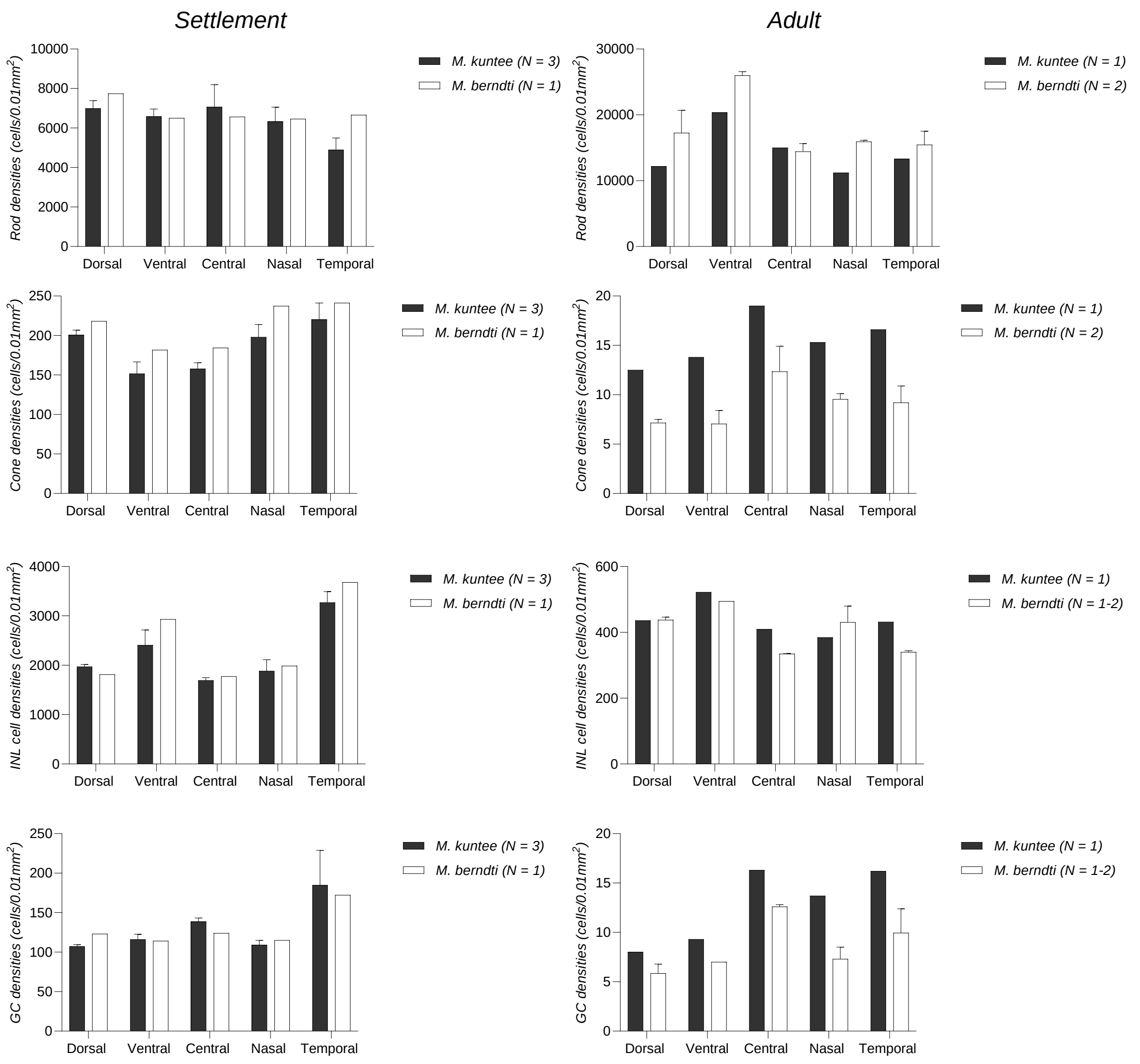

Fig. S1. Comparison of retinal cell densities in two species in Myripristinae. Graphs showing that Abercrombie-corrected cell densities are similar between *Myripristis kuntee* and *M. berndti* for each stage and retinal region. INL, inner nuclear layer; GC, ganglion cell.

Journal of Experimental Biology • Supplementary information

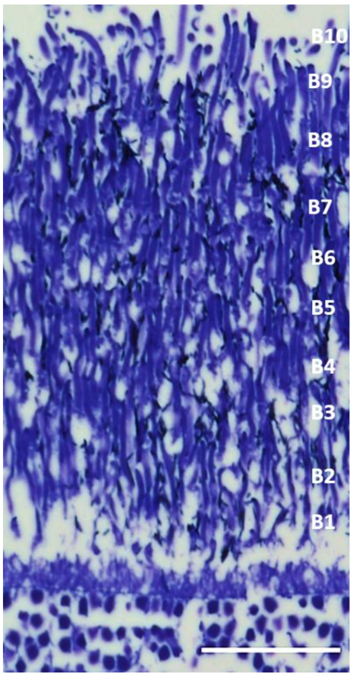

Fig. S2. The multibank retina in *Ostichthys* sp. Representative transverse section of the ventral retina showing photoreceptor layer with multiple banks of rods (up to approximately 10) in an adult deep-dwelling soldierfish (*Ostichthys* sp). Banks are numbered $B_{1\text{-}n}$. Scale bar: 30 µm.

Table S1. Details of animals used in study. This study used a total of 20 individuals from the family Holocentridae, 18 of which were collected in the current study and 2 of which were collected in de Busserolles *et al.* (2021). Locations: LI, Lizard Island; MI, Moorea Island; CM, Cairns Marine. Analyses: Cell densities, cell densities in each retinal region quantified; Histology, rod banking examined in histological sections. Standard length was not recorded for one individual and therefore, is marked as n.a.

Species	Life stage	Standard length (cm)	Location	Eye used	Analyses performed	Reference
Sargocentron rubrum	Pre-settlement larva	3.1	LI	R	Histology, Cell densities	This study
	Pre-settlement larva	2.6	LI	L	Histology, Cell densities	This study
	Pre-settlement larva	2.8	LI	R	Histology, Cell densities	This study
	Settled juvenile	3.4	LI	L	Histology, Cell densities	This study
	Settled juvenile	3.2	LI	L	Histology, Cell densities	This study
	Adult	13.4	CM	L	Histology, Cell densities	This study
	Adult	14.0	LI	R	Histology, Cell densities	(de Busserolles *et al.* 2021)
	Adult	n.a.	LI	R	Histology, Cell densities	This study
Myripristis kuntee	Settlement larva	6.5	MI	R	Histology, Cell densities	This study
	Settlement larva	5.2	MI	L	Histology, Cell densities	This study
	Settlement larva	5.6	MI	R	Histology, Cell densities	This study
	Adult	14.4	MI	R	Histology, Cell densities	This study
Myripristis berndti	Settlement larva	4.9	MI	L	Histology, Cell densities	This study
	Adult	17.7	CM	R	Histology, Cell densities	This study
	Adult	17.5	CM	R	Histology, Cell densities	This study
Ostichthys sp.	Adult	20.5	MI	L	Histology	This study
Sargocentron microstoma	Settlement larva	5.8	MI	R	Histology	This study
	Adult	13.5	MI	R	Histology	This study
Sargocentron diadema	Adult	10.0	LI	L	Histology	(de Busserolles *et al.* 2021)
Myripristis violacea	Adult	14.4	MI	R	Histology	This study

References

de Busserolles, F., Cortesi, F., Fogg, L., Stieb, S. M., Luehrmann, M., and Marshall, N. J. 2021. 'The visual ecology of Holocentridae, a nocturnal coral reef fish family with a deep-sea-like multibank retina', *J Exp Biol*, **224**, jeb233098.

Journal of Experimental Biology • Supplementary information
